# Biomarker changes in suspected idiopathic normal-pressure hydrocephalus patients undergoing external lumbar drainage: a pilot study

**DOI:** 10.3325/cmj.2024.65.328

**Published:** 2024-08

**Authors:** Klara Brgić Mandić, Goran Mrak, Hrvoje Barić, Sergej Marasanov, Goran Šimić, Ena Španić Popovački, Marijan Klarica

**Affiliations:** 1 Department of Neurosurgery, University Hospital Center Zagreb, Zagreb, Croatia; 2 Department of Neuroscience, Croatian Institute for Brain Research, University of Zagreb School of Medicine, Zagreb, Croatia; 3 Department of Pharmacology and Croatian Institute for Brain Research, University of Zagreb, School of Medicine, Zagreb, Croatia; Brgić Mandić et al: Biomarker changes in suspected idiopathic normal-pressure hydrocephalus patients

## Abstract

**Aim:**

To examine whether changes in biomarker concentrations in patients with idiopathic normal-pressure hydrocephalus (iNPH) during 72 h of external lumbar drainage (ELD) can differentiate between responders and non-responders.

**Methods:**

Twenty patients with clinical and neuroradiological signs of iNPH underwent ELD over a period of 72 h. During this period, changes in cerebrospinal fluid (CSF) concentrations of biomarkers (amyloid-β, total and phosphorylated tau proteins) and intracranial pressure were monitored, and the volume of drained CSF was measured. Changes in the concentrations of selected biomarkers at three time points (0, 36, and 72 h) during ELD were tested for association with changes in clinical condition.

**Results:**

Ten patients showed significant clinical improvement after ELD, quantified as a difference of two or more points on the Mini-Mental State Examination and/or Japanese iNPH grading scale. The concentration of all tested biomarkers increased during the first 36 h. Respondents had higher Aβ 1-42 at all time points, with a significant difference seen after 72 h. They also had a significantly higher Aβ1-42/Aβ1-40 ratio at all time points.

**Conclusion:**

A gradual increase in Aβ 1-42 concentration during three-day ELD represents a possible positive prognostic factor for the placement of permanent CSF drainage in patients with iNPH.

 Idiopathic normal-pressure hydrocephalus (iNPH) is characterized by a triad of symptoms: dementia, gait disturbances, and urinary incontinence (1). Gait disturbances are often the first symptom, present in 94%-100% of patients at diagnosis (2-9). Cognitive impairments are observed in 78%-98% of patients, while urinary urgency and incontinence affect 60%-92% at diagnosis (4,8). The disorder is believed to result from the pressure of enlarged ventricles on motor fibers in the corticospinal pathway (10). The prevalence of iNPH in people older than 65 years is 3.7% (11).

The most common differential diagnosis for iNPH is Alzheimer disease (AD), the most prevalent neurodegenerative condition (12-14). Up to 75% of patients exhibit pathohistological characteristics of both AD and iNPH (14-18). For AD diagnosis, three core biomarkers are crucial: amyloid beta 1-42 (Aβ 1-42), total tau (t-tau), and phosphorylated tau protein (p-tau) (19,20). The full-length Aβ 1-42 is extremely hydrophobic and forms oligomers and fibrils that accumulate in extracellular plaques, which are characteristic of AD (17). Presumably due to the accumulation of Aβ 1-42 in plaques, its concentration in cerebrospinal fluid (CSF) is notably lower in AD patients than in the healthy population (21-26). Although in patients with iNPH, amyloid-β concentration tends to be lower than in the healthy population, these values are still higher than those in patients with AD (27). Patients with iNPH have a higher concentration of total tau protein (28), p-tau protein, and amyloid precursor protein (APP) and its fragments in CSF than the healthy population, but lower than AD patients (29). Another useful marker in discriminating iNPH from AD may be CSF phosphorylated tau protein at threonine position 181 (pT181), alone or in combination with total tau (30,31).

Factors determining CSF movement along the CSF system, which affect its interaction with interstitial fluid, certainly influence the fate of molecules of different molecular weight and their distribution between tissue and CSF (32-34). Healthy people, for instance, have a higher concentration of monoamines and their metabolites in cranial than in lumbar CSF, a finding that challenges the traditional view of a unidirectional CSF circulation (35,36). This suggests a more complex interaction between CSF and brain tissue metabolism. When 10 mL (37) to 15 mL (38) of lumbar CSF was sampled, the concentration of various monoamines exponentially increased in individual fractions of the CSF sample (first, middle, and last milliliters). This observation indicates that CSF closer to the brain tissue contains a higher concentration of monoamines and their metabolites, contrary to an even distribution expected according to the classic concept of one-way CSF circulation, reflecting changes in the metabolism of adjacent tissue (37). In addition, in CSF samples obtained by free cisternal drainage in animals over two hours, the concentration of monoamine metabolites exponentially increased, which indicated an influx of CSF from higher parts containing higher concentrations of the measured substances (39).

Contrary to the concentrations of monoamines within the CSF system, the concentrations of blood-derived proteins in the ventricle are lower compared with the lumbar compartment (40,41). When extracting CSF through lumbar puncture in healthy people, the protein concentration in CSF decreases in subsequent fractions (37). This observation additionally indicates that CSF is not mixed by circulation. Moreover, extracting a CSF sample from higher parts with a lower protein concentration can easily explain the observed phenomenon of protein concentration drop in lumbar CSF in later fractions.

iNPH is characterized by biochemical changes in CSF that reflect metabolic changes in the brain. CSF is in direct contact with the extracellular space and is therefore considered a good source of potential biomarkers. Similar to the concentration gradient for proteins in the CSF system, there also appears to be a concentration gradient for peptides such as Aβ 1-42 (42). The preoperative concentration of Aβ 1-42 in the lumbar region was shown to be higher than the postoperative concentration in the ventricles (42).

In contrast to healthy people, patients with AD and iNPH are expected to have lower Aβ 1-42 levels in initial CSF samples due to amyloid accumulation in the interstitial space. Specifically, during preoperative testing, patients with suspected iNPH undergoing prolonged external lumbar drainage (ELD) with larger CSF volumes drained, are expected to have an increased peptide biomarker concentration. This is because low CSF pressure may induce hydrostatic drawing of water from the blood into the interstitial space and CSF, resulting in the “washing out” of accumulated Aβ 1-42 (43,44). We hypothesized that changes in peptide concentration in the CSF of patients evaluated for potential iNPH management differed from those observed previously in healthy people. Furthermore, we postulated that Aβ concentration from the onset to the end of prolonged CSF drainage, due to suspected iNPH, varied between responders and non-responders. Consequently, we assessed AD biomarker concentrations in collected CSF samples during extended ELD immediately after placement, and after 36 and 72 hours, while monitoring changes in CSF pressure, volume of drained CSF, and clinical response.

## Patients and methods

### Patients

This study was conducted at the University Hospital Center Zagreb from February 2018 to April 2023. The research was approved by the Ethics Committee of the University Hospital Center Zagreb and the Ethics Committee of the University of Zagreb School of Medicine.

The study enrolled patients who underwent drainage placement due to suspected iNPH manifesting as cognitive impairment, gait disturbances, and urinary incontinence. To quantify cognitive impairment, the Mini-Mental Status Examination (MMSE) was used. The Japanese iNPH scale (JiNPHS) was used to quantify other symptoms. A MMSE score of less than 24 indicated significant cognitive deterioration (45).

**Figure 1:**
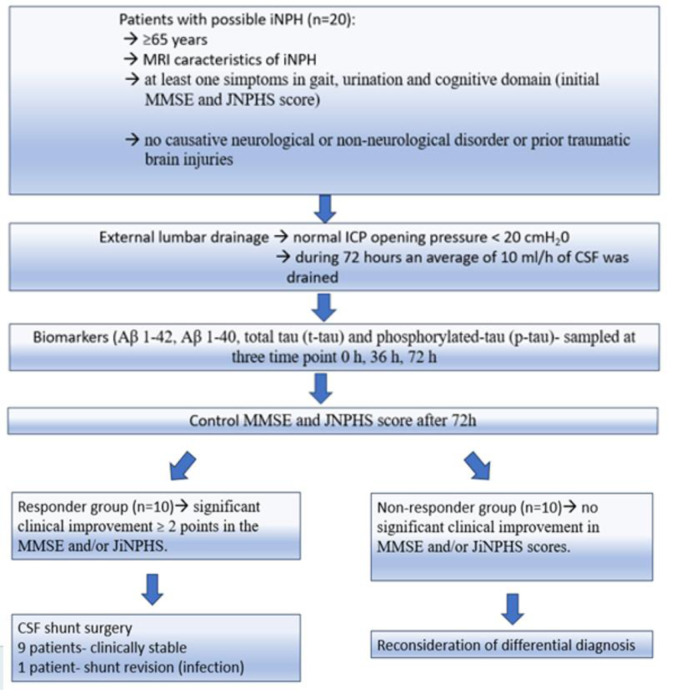
Flow diagram of patient selection. MRI – magnetic resonance imaging; iNPH – idiopathic normal-pressure hydrocephalus; MMSE – Mini-mental State Examination; JiNPHS – Japanese iNPH Scale; CSF – cerebrospinal fluid.

Patients were required to meet the neuroradiological criteria of iNPH, including ventriculomegaly, especially of the frontal and temporal horns, Evans index >0.3, bulging of the corpus callosum more cranially, callosal angle <90°, significant expansion of the Sylvian fissure disproportionate to the expansion of the convexity sulcus (especially in the parietal region), and increased signal in T2-measured periventricular MRI sequences. In patients with clinical and radiological suspicion of iNPH, standard testing involved placing an external lumbar drain and draining a large amount of CSF ( ~ 10 mL/h) over a 72-hour period. The exclusion criteria were underlying neurological or non-neurological disorders that can cause the same symptoms and a history of severe brain trauma. The opening CSF pressure during ELD placement had to be within reference values, meaning ≤20 cm H_2_O. Before drainage placement, patients were thoroughly evaluated for the severity of clinical symptoms (MMSE and Japanese iNPH scale). A follow-up clinical evaluation was performed after 72 hours (Figure 1). Patients who had shunt surgery were followed up at 1-month and 6-month intervals postoperatively. Nine patients remained clinically stable, while one required shunt revision due to infection.

### Method of CSF pressure recording during ELD

Lumbar drainage was placed between the intervertebral space L_IV_/L_V_ or L_V_/S_I_, depending on the anatomical predisposition of the patients, and was determined in relation to the iliac crest. The puncture site was prepared in the standard way, and a 14-16 G Tuohy needle was inserted. After removing the stylet, the needle was connected to the monitor via a transducer placed at the level of the internal auditory canal (IAC), and the first lumbar opening pressure was measured. Subsequently, a lumbar drain (Medtronic EDM Lumbar Drainage Kit, Minneapolis, MN, USA) was placed through the needle and connected to the transducer and the CSF collector via the T connection. CSF pressure was monitored hourly in the horizontal position, corresponding to intracranial pressure when the pressure transducer is positioned at the level of the IAC.

### Quantification of biomarkers in CSF

Immediately after ELD placement, 1-2 mL of CSF was sampled for biomarker quantification (β-amyloid proteins [Aβ 1-42, Aβ 1-40], total tau [t-tau], and phosphorylated-tau [p-tau]). Sampling was repeated after 36 h and 72 h. CSF samples were stored in Eppendorf pure polypropylene tubes (Eppendorf, Merck KGaA, Darmstadt, Germany). After centrifugation at 2000 × g for 10 min, CSF samples were stored at -80 °C until further analysis. The biomarker levels in CSF were determined with enzyme-linked immunosorbent assay according to the manufacturer's protocols: Aβ1-42 with Innotest β-amyloid 1-42; total tau with Innotest hTau Ag; and p-tau181 with Innotest Phospho-Tau (181P) (all by Fujirebio, Gent, Belgium).

Since biomarker concentrations can sometimes vary significantly (46), each sample was analyzed in duplicate. Each biomarker was measured on the same day using the same batch of reagents. If the measured concentrations of a sample differed by more than 10%, the measurement was repeated. All reported concentrations are the mean values of two measurements.

### Statistical analysis

Continuous variables are presented as median (range or interquartile range), and nominal variables as absolute (relative) frequency. Differences between the groups in continuous variables were assessed with the Mann-Whitney U test. Differences between the groups in nominal variables were assessed with the χ^2^ test. The before- and after-ELD placement comparisons were performed with the Wilcoxon signed-rank test. Multiple comparisons of biomarker levels across time were performed with the Friedman test. All tests were two-sided. The level of statistical significance was set at *P* = 0.05. SPSS, version 25.0 (IBM Corp., Armonk, NY, USA), and GraphPad Prism, version 8.4.3 (GraphPad Software, Boston, MA, USA), were used for statistical analysis and graphical presentation of the results.

## Results

A total of 20 patients with suspected iNPH underwent testing. Sixty percent were female, with a median age of 72.5 years (Table 1). This aligns with global findings that iNPH predominantly affects individuals over 65 years of age. MMSE scores significantly increased, and JiNPHS scores significantly decreased after ELD testing (the Wilcoxon signed-rank test) (Table 2).

**Table 1:** Sociodemographic and clinical data for responder and non-responder groups. Numbers are median (range) or absolute (relative) frequency*

	Responders (n = 10)		Non-responders (n = 10)		P
	median	range	median	range	
Age (years)	71	65-82	74	67-82	0.393
Sex (female/male)	5/5	50/50	7/3	70/30	0.650
MMSE before ELD	27	21-28	24	4-29	0.280
MMSE after ELD	29	23-30	26	4-30	0.143
JiNPHS before ELD	5	3-7	4	3-11	>0.999
JiNPHS after ELD	2	1-5	4	1-11	0.015
CSF volume drained in 72 h (mL)	730	570-931	787	650-982	0.278

*Abbreviations: MMSE – Mini-Mental State Examination; JiNPHS – Japanese iNPH Scale; ELD – external lumbar drainage, CSF – cerebrospinal fluid.

**Table 2:** Comparisons of clinical scores before and after external lumbar drainage placement

		N	Mean rank	Sum of ranks	*P* value
Mini-mental State Examination	negative ranks	0	0	0	<0.001
	positive ranks	16	8.50	136.00	
	ties	4			
	total	20			
Japanese Idiopathic Normal-Pressure Hydrocephalus Scale	negative ranks	13	7.00	91.00	0.001
	positive ranks	0	0	0	
	ties	7			
	total	13	7.00	91.00	

Out of all patients tested for suspected iNPH, 10 showed significant clinical improvement after ELD (responders), quantified as a difference of two or more points in either MMSE or JiNPHS. Over three days, the average CSF volume drained was 770 mL, ranging from 570 to 982 mL. In the responder group, an average of 730 mL of CSF was drained over 72 hours, compared with 787 mL in the non-responder group (Table 1).

The average opening pressure during ELD placement was 6.5 cm H_2_O in both groups. After 36 h, the median pressure in the responder group increased to 7.5 cm H_2_O, while it averaged 5 cm H_2_O in the non-responder group. The pressure difference between the groups decreased after 72 h (6 cm H_2_O and 5 cm H_2_O, respectively).

**Figure 2:**
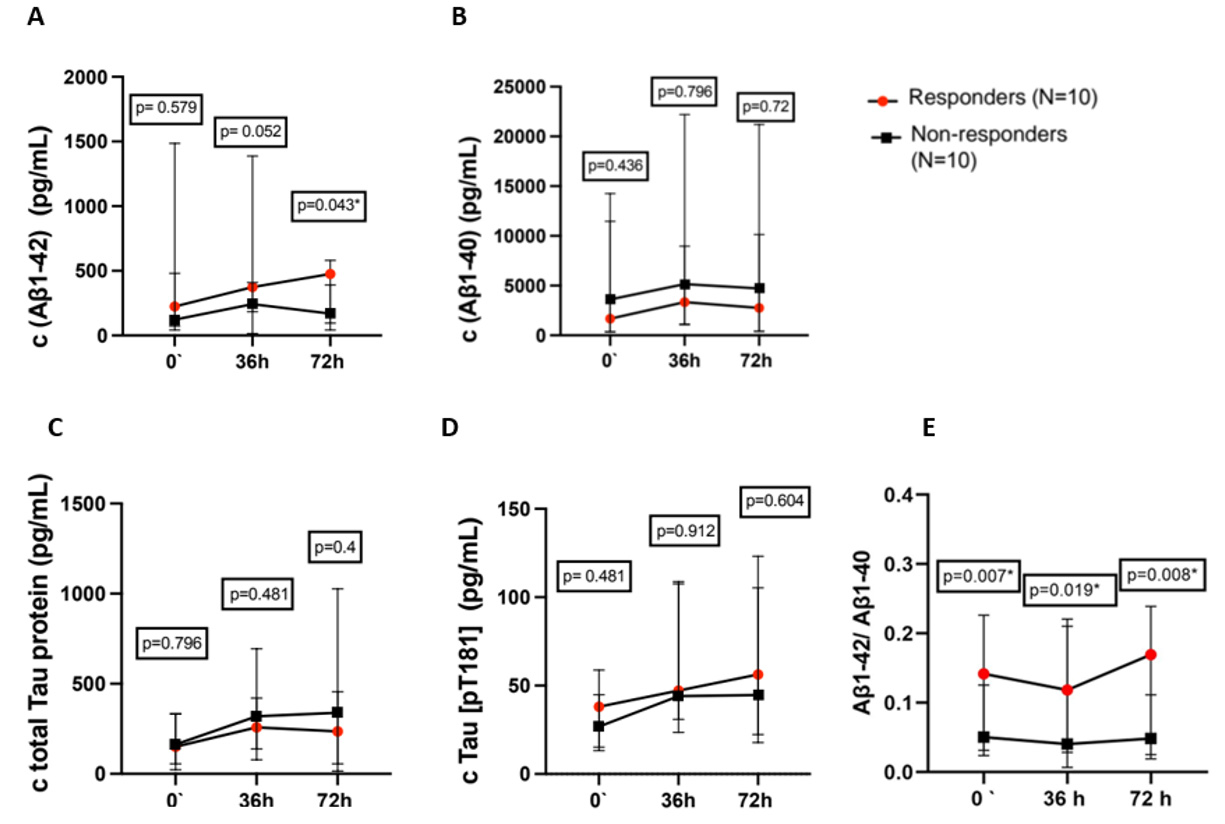
Concentrations of different biomarkers in the responder and non-responder groups: (**A**) amyloid Aβ1-42, (**B**) Aβ1-40, (**C**) total tau protein (t-tau), (**D**) phosphorylated tau protein (pT181) (expressed in pg/mL), and (**E**) Aβ1-42 /Aβ1-40 ratio in cerebrospinal fluid at time points: 0`– immediately after external lumbar drainage placement, after 36 hours, and after 72 hours of drainage; * significant difference.

The levels of Aβ1-42, Aβ1-40, total tau, and pT181 biomarkers significantly changed over time, with the exception of the Aβ1-42/Aβ1-40 ratio (Table 3). Aβ 1-42 levels were higher in responders compared with non-responders at all measured points, with a significant difference noted only after 72 h (Figure 2A, Table 4). Although non-responders had higher total tau (Figure 2C) and Aβ 1-40 levels (Figure 2B) at all time points, the differences were not significant. Similarly, pT181 levels were higher in the responder group at all times, but the difference did not reach significance (Figure 2D). Responders had a significantly higher Aβ1-42/Aβ1-40 ratio at all time points (Figure 2E, Table 4).

**Table 3:** Concentrations of amyloid Aβ1-42, Aβ1-40, total tau protein phosphorylated tau protein (pT181), and amyloid Aβ1-42/Aβ1-40 ratio in cerebrospinal fluid immediately after external lumbar drainage placement (0`), after 36 hours, and after 72 hours of drainage

	0`		36 h		72 h		*P*
	median	range	median	range	median	range	
Aβ1-42 (pg/mL)	144.47	42.86-1486.14	320.02	15.24- 1386.78	273.93	42.86-580.41	0.010
Aβ1-40 (pg/mL)	2531.05	341.13-14250.56	4181.17	1064.94-22197.72	4254.3	384.28-21188.43	0.027
Aβ1-42/Aβ1-40 (pg/mL)	0.08424	0.02381-0.22653	0.06166	0.00686-0.22069	0.05726	0.01927-0.23922	<0.911
Total tau	161.2	22.69-334.99	275.8	77.51-693.76	296.33	15.77-1027.10	<0.001
Tau pT181	27.42	13.14-58.86	50.92	23.57-108.78	48.74	17.83-123.23	<0.001

**Table 4:** Concentrations of amyloid Aβ1-42, Aβ1-40, total tau protein, phosphorylated tau protein (pT181), and amyloid Aβ1-42 /Aβ1-40 ratio in cerebrospinal fluid in the responder and non-responder groups immediately after external lumbar drainage placement (0`), after 36 hours, and after 72 hours of drainage

	Responders (n = 10)		Non-responders (n = 10)		P
	median	range	median	range	
Aβ1-42 (pg/mL)					
0`	224.535	71.99-1486.14	122.285	42.86-481.24	0.579
36 h	373.5	183.8-1386.78	242.435	15.24-409.89	0.052
72 h	476.52	96.19-580.41	168.755	42.86-391.52	0.043
Aβ1-40 (pg/mL)					
0`	1668.76	384.28-14250.56	3621.33	341.13-11462.74	0.436
36 h	3336.72	1137.97-22197.72	5164.27	1064.94-8969.21	0.796
72 h	2756.75	424.6-21188.43	4721.04	384.28-10134.47	0.720
Aβ1-42/Aβ1-40					
0`	0.14183	0.03153-0.22653	0.05037	0.02381-0.12564	0.007
36 h	0.11842	0.02858-0.21062	0.04065	0.00686-0.22069	0.019
72 h	0.16955	0.02529-0.23922	0.04845	0.01927-0.11153	0.008
t- tau (pg/mL)					
0`	150.85	55.72-334.99	163.24	22.69-331.34	0.796
36 h	257.905	138.1-420.49	319.77	77.51-693.76	0.481
72 h	235.9	55.72-455.39	339.1	15.77-1027.1	0.400
pT181 (pg/mL)					
0`	38.05	13.14-58.86	26.77	15.21-44.77	0.481
36 h	47.15	30.90-108.78	44.06	23.57-107.53	0.912
72 h	56.26	17.83-105.27	44.71	22.31-123.23	0.604

## Discussion

In our study, the concentration of all tested biomarkers increased within the first 36 hours after drainage placement. This finding suggests that significant CSF volume drainage induces a continuous influx of these substances from tissues (where they accumulate) into CSF. This phenomenon, previously observed during hourly CSF collection over 36 h (47), remains unexplained.

Aβ 1-42 concentration consistently rose in the responder group, which indicates that continuous CSF drainage facilitates the washout of accumulated Aβ 1-42 from the interstitial space into CSF. Given that Aβ 1-42 deposits predominantly in the gray matter, an increase in lumbar CSF Aβ 1-42 concentration likely results from CSF arriving from cranial regions with higher Aβ 1-42 concentrations. After 72 hours, Aβ1-42 levels were significantly higher in the responder group, which suggests this could be a new prognostic factor for surgery if confirmed in larger studies.

CSF biomarkers are used to differentiate patients likely to show clinical improvement after the placement of a permanent shunt from those mimicking iNPH symptoms. Leinonen et al demonstrated that in 22% of patients with suspected iNPH, the presence of amyloid plaques and neurofibrillary tangles in biopsy samples correlated with AD development over 4.4 years (18). AD is characterized by an increased concentration of total and phosphorylated tau proteins and a decreased concentration of Aβ 1-42 (20,21). A meta-analysis of 13 studies (48-60) showed that shunt-responder patients had lower lumbar CSF concentrations of total and phosphorylated tau proteins than non-responders, with no significant difference in Aβ 1-42 levels (61). However, the generalizability of these results is limited due to variations in analytical methods, methodological weaknesses of the studies, the small number of studies dealing with Aβ 1-42 changes, and differing CSF sample collection times after varying drainage durations. Tarnaris et al demonstrated, in 11 patients with suspected iNPH, that the concentrations of Aβ1-42 and total tau protein increased during 72 h of CSF evacuation via ELD (62). Jingami et al found that during the tap test, there was an increased concentration of total tau protein and decreased levels of Aβ 1-42 in the last milliliter compared with the first milliliter of CSF (63). Considering that the volume of drained CSF in the cited study was 30 mL, these results cannot be directly compared with ours, where the drained CSF volume was considerably higher (770 mL).

Our findings demonstrate changes at three time points (0', 36 h, 72 h) during ELD of a significant total CSF volume (average 770 mL under lower CSF pressure). In the responder group, the concentration of the Aβ 1-42 isoform was consistently higher at all time points compared with the non-responder group, with the difference reaching significance only after 72 h. This observation, under these specific conditions, significantly differs from those of previous studies. Additionally, in our study, the non-responder group had higher total tau protein and Aβ 1-40 at all time points, though without reaching statistical significance. In the responder group, the concentration of total tau protein decreased after 72 h, which indicated that following the initial washout of this marker characteristic of neurofibrillary neurodegeneration, its concentration in patients without AD comorbidity in the lumbar CSF sample stabilizes. Higher values of pT181 were also observed in the responder group at all time points, but without a significant difference, which contrasts with the findings of previous studies, in which responders had lower values of pT181.

Another longitudinal study, tracking the concentrations of biomarkers in the lumbar and ventricular spaces over time, showed that in patients with probable iNPH and a negative Aβ brain biopsy, the concentration of Aβ 1-42 shifted toward more positive values compared with patients with iNPH and a positive Aβ brain biopsy, particularly after a longer follow-up. This indicates that, in patients with probable iNPH, a higher Aβ 1-42 value acts as the best negative predictor of underlying AD (64). These findings support the notion of a better clinical response to permanent shunt placement in patients with higher Aβ 1-42 levels in the lumbar CSF, aligning well with our results.

According to the new concept of CSF physiology, CSF can be produced at the level of the brain and spinal tissue capillary network if an osmotic or hydrostatic gradient is created, which facilitates the entry of a net volume of fluid from the capillaries into the interstitial space and CSF (43,44). During ELD, the reduction of hydrostatic CSF pressure to 5-7.5 cm H_2_O enables the entry of fluid from the capillary network into the interstitial space of the cerebral gray matter and potentially facilitates the “washout” of substances accumulated in the interstitium into the CSF system. This mechanism may explain the observed gradual increase in Aβ 1-42 concentration during prolonged ELD in responders.

The current study is subject to several major limitations, including a small sample size, single-center design, and the absence of long-term follow-up data. These factors limit the generalizability of our findings and the ability to assess how biomarker changes correlate with long-term outcomes, such as the progression of neurological symptoms and mortality. Additionally, the lack of a control group to directly compare the biomarker levels may also affect the interpretation of our results. Without a control group, it is challenging to ascertain whether the observed biomarker changes are specific to the treatment received or reflect natural disease progression. Another potential limitation is the variability in the diagnostic criteria for iNPH across different centers, which may introduce selection bias and affect the applicability of our findings to a broader population. Furthermore, we did not perform a brain biopsy, which would definitively determine the coexistence of an underlying neurodegenerative disease. Finally, our study did not account for potential confounding factors such as variations in patients' medication use, comorbid conditions, or lifestyle factors that could influence biomarker levels. Addressing these limitations in future research is essential for a more comprehensive understanding of the implications of biomarker changes in patients with iNPH.
